# Current Topics

**Published:** 1892-05

**Authors:** 


### CURRENT TOPICS.

THE State of Kentucky, always loyal to legitimate medicine, has a most novel and satisfactory way of dealing with traveling or itinerant doctors who pretend to practise within its boundaries. We learn from the Cincinnati *Lancet-Clinic* that the City of Bowling Green, Ky., has passed an ordinance setting forth that it shall be unlawful for any traveling or itinerant doctor to practise medicine in any of its branches within the limits of the State. The penalty for the violation of the ordinance is from $50 to $100 for each day of such violation. Similar ordinances have been passed in Lexington, Paducah, Harrisburg, Lebanon, Stanford, Franklin, Glasgow, Elizabethtown, and several other cities, and is reported to be pending, with the probability of passing in nearly every other city and town in the State of Kentucky. We commend the idea to other states that are afflicted with these parasites.

---

THE OHIO MEDICAL PRACTICE ACT, that has been pending in the State Legislaturese, ems to have been strangled by a combination of quacks and irregulars, who have overwhelmed the better element in Ohio. We are not surprised at this defeat, because we have had a similar experience in our own State. It took us many years to convince the Legislature that it would be wise for the State to assume the right to grant license to physicians independent of medical colleges, and in the struggle we met not only the opposition of the quacks, itinerant pill peddlers, and other human vermin, but we are sorry to say that some of the most orthodox, the most ethical (?), and the most solid members of the profession in the State were detected in secretly conniving to prevent the passage of such a measure. Year after year, however, we crossed swords with them in the halls of the capitol, in committee rooms, in medical societies, and in the clubs, until we finally overwhelmed them, and have succeeded in obtaining a very complete and efficient law. More 's the pity, however, that we have been thwarted in carrying out its provisions for a year or two, by the combinaton of medical students who have succeeded in obtaining exemptions from this statute. The medical profession, from one end of the State to the other, has joined hands with New York, Brooklyn, and Buffalo, in endeavoring to check the pernicious legislation referred to, with what effect at this writing we know not, but we hope for the best. Meanwhile, we feel sure that the classes of '93 in this State will be subjected to the operations of the law.

The Food Manufacturers' Association has introduced a novelty with reference to its proposed exposition, to be held in Madison Square Garden, in October next. We refer to the engagement of the Seidl Orchestra, which, under the direction of Herr Anton Seidl, is to conduct afternoon and evening concerts during the entire time of the exposition. This will afford the public an opportunity to hear most delightful music, and thus will be combined the esthetics and the substantials of life in a most novel and acceptable manner.

---

Dr. C. H. Mastin, of Mobile, President of the American Surgical Association, in his address delivered in Washington in September, 1892, paid an eloquent tribute to the late Dr. Samuel David Gross, and recommended that the association should take upon itself to initiate a movement for the erection of a suitable monument to perpetuate the memory of this most distinguished American surgeon.

The American Surgical Association does not propose to monopolize the honor of this grand project, but it appeals to the entire American profession to come forward with contributions and with good will to enable it to carry out its design. Committees and sub-committees have been appointed, and Dr. John B. Roberts, 1627 Walnut street, Philadelphia, has been chosen treasurer ; all contributions, therefore, should be sent to him.

We need not do more than to call the attention of the profession to this subject, for we feel sure that it is one that will appeal to its sense of propriety without further urging on our part. The amount needed to erect an imposing statue in bronze, of heroic size, upon a granite pedestal, the whole being about sixteen feet in height, will not exceed $12,000. The committee now only requires a little more than $8,000, and it would seem to us that this amount should be promptly subscribed.

---

The first decennial catalogue of the College of Physicians and Surgeons, of Chicago, has lately come to our table. It is by far the handsomest college announcement and catalogue that we have seen, and might be adopted as a model by other American colleges with great propriety. The fine photographic reproductions of the college building and Cook County Hospital, that appear as frontispieces, introduce the book, which consists of eighty-two duodecimo pages carefully indexed, in a most favorable way; but particularly do we note that the requirements for admission to this college are of the most satisfactory character. These requirements are plainly and distinctly named in unmistakable terms, and it affords us much pleasure to say that among the equivalents of the preliminary education which this college expects is the certificate of the Regents of the University of the State of New York. The course of study is definitely planned, and covers four years, each year to consist of a Winter term of twenty-six weeks, with the usual Spring term addendum of twelve weeks, which latter is optional.

Among the requirements for graduation we note the following: "(3.) Four full years of study of medicine under the direction of a physician or medical college recognized by the Illinois State Board of Health as such." Surely the degree of the College of Physicians and Surgeons of Chicago may be well worth contending for, and when obtained it will be considered throughout the land as evidence of ample equipment by the young physician.

---

THE Toronto General Hospital has lately issued its annual report for the year ending September 30, 1891. It is a handsome brochure of 107 pages, illustrated with lithographs and woodcuts of the hospital buildings as they formerly appeared, and as they now look with the improvements recently made upon them. Besides the ordinary record of cases treated and their several classifications, there are divisions of the report relating to medical education, history of the hospital, description of the hospital buildings, and a roster of the trustees from 1853 to the present. The ambulance service is given in detail with illustrations, and, finally, the annual report of the training school for nurses is added. Taken all together, this is one of the most complete and useful hospital reports that we have seen, and reflects much credit upon all concerned in that useful institution, but especially upon the medical superintendent, Dr. Chas. O'Reilley, who has held the position for more than sixteen years.

It seems to us that this is the proper way to govern a hospital, in order that its patients may receive the full benefit of the most skilful attention. A medical superintendent who is fitted for the work will be obeyed and respected when others will not, and thus insure perfect subordination in all the various departments.

THE *American Journal of Obstetrics* for April contains the following official announcement:

The Section on Gynecology and Abdominal Surgery at the Pan-American Medical Congress has been organized by the election of Dr. William Warren Potter, 284 Franklin street, Buffalo, N. Y., as Executive Chairman; Dr. Brooks H. Wells, 71 West 45th street, New York City, as English-speaking Secretary; and Dr. Ernest W. Cushing, 168 Newberry street, Boston, as Spanish-speaking Secretary. The Foreign Secretaries of the Section so far selected are: *The Argentine*, Dr. Dn. L. C. Maglioni Llobet, Victoria 737, Buenos Ayres; *Brazil*, Dr. Dn. Luiz da Cunha Feiho, Rio de Janeiro; *British North America*, Dr. Jas. F. W. Ross, 481 Sherborne street, Toronto, Canada; *Columbia*, Dr. Dn. José W. Buendia, Calle 10, No. 212, Bogota; *Nicaragua*, Dr. Juan I. Urtecho, Calle Real, Ciudad Granada; *Spanish West Indies*, Dr. Dn. Gabriel Casuso, Virtudes 37, Habana, Cuba; *Uruguay*, Dr. Dn. Enrique Peréy, Uruguay 371, Montevideo.

---

LAST month we called attention to the fact that Dr. E. E. Montgomery had resigned the Obstetrical Chair in the Medico-Chirurgical College for the purpose of devoting himself exclusively to teaching in the gynecological department of that college. We have in another place alluded to the fact that Dr. Montgomery has since been appointed Clinical Professor of Gynecology in Jefferson Medical College, and hence severs his connection with the Medico-Chirurgical institution. The Board of Trustees of Jefferson Medical College have also established the following clinical professorships, electing Dr. F. X. Dercum, Professor of Nervous Diseases; Dr. E. E. Graham, Professor of Children's Diseases; Dr. H. Augustus Wilson, Professor of Orthopedic Surgery; Dr. H. W. Stelwagon, Professor of Dermatology, and Dr. W. M. L. Coplin, Adjunct Professor of Hygiene.

---

MESSRS. CHARLES TRUAX, GREENE & CO. propose to establish a bureau of service and information to be operated by them during the entire session of the World's Fair free of all expense to the medical profession. They offer their services in regard to obtaining hotels, and boarding houses, the receipt and delivery of telegrams, postal facilities, banking conveniences, together with many other attentions that relate to the entertainment of visitors during the Columbian Exposition. The services connected with this bureau are entirely free to the medical visitors in attendance at the fair, and is another indication of the many generosities of the Chicago inhabitants. It will, no doubt, be a great convenience to the profession, and will be largely visited during the season.

---

The *International Medical Magazine*, edited by Dr. Judson Daland, and published by J. B. Lippincott Co., Philadelphia, is a handsome large octavo of over 100 pages, which number first made its appearance in February of the present year. Among its other many interesting features, it has established a medico-legal department, in which it will deal with the legal responsibilities incident to the practice of medicine and surgery, that will prove of interest and value to the general practitioner and specialist. Taken all in all, this is one of the handsomest printed and most systematically arranged of all the general medical magazines that we have seen, and we feel assured that it will command a large patronage almost at once.

---

A special examination in medicine will be held at 21 Cooper Union, New York City, May 3–6, 1892, to accommodate candidates for license to practise in this State. The examination will be conducted in accordance with the usual daily programme, and will be held in New York City only. Candidates who desire admission to this examination must file application blanks not later than Saturday, April 30th. The next regular examination will be held June 14–17, 1892.

---

Papoid, or Vegetable Pepsin, is now becoming recognized as a medicine of value, and will, undoubtedly, take its place in the official list very soon. It is manufactured by the Papoid Company, Johnson & Johnson, Sole Agents, 92 William St., New York, who have recently issued a most interesting pamphlet containing information as to the methods of administering papoid, and many other facts that have been gleaned from American and foreign literature and grouped in a single brochure, very convenient for reference.

---

The Graduating Exercises of the Medical Department, Niagara University, will take place Monday evening, May 2d, at the Star theatre. Exercises begin at 8 o'clock p. m.

